# Judith Butler’s theoretical perspectives within a nursing context—a scoping review

**DOI:** 10.1177/09697330241257569

**Published:** 2024-06-05

**Authors:** Adelheid Hummelvoll Hillestad, Eline Kaupang Petersen, Maud C Roos, Maria H Iversen, Trine Lise Jansen, Monica Evelyn Kvande

**Affiliations:** 155319 Lovisenberg Diaconal University College; 60504 University of Oslo; 177041 University of South-Eastern Norway (USN); 155319 Lovisenberg Diaconal University College

**Keywords:** Ethics, Judith Butler, nursing, scoping review, vulnerability

## Abstract

Philosopher Judith Butler has influenced how people talk about vulnerable bodies and sees vulnerability as universal, existential, and relational. Being vulnerable is part of the human condition. The main theoretical areas that run across Butler’s work; power, knowledge and subjectivity, performativity, and ethics—are of particular relevance to nursing practice. This review aims to explore how Butler’s theoretical work is reflected in research literature within a nursing context. We conducted a scoping review guided by Arksey and O’Malley’s methodological framework. A systematic literature search of CINAHL (EBSCOhost), MEDLINE (Ovid), Embase (Ovid), PsycINFO (Ovid), and Web of Science identified 15 papers. Butler’s theoretical work was applied at an individual and social level in research literature within a nursing context. Nurses need to reflect on their clinical practice and role as health professionals in relation to power and performativity in encounters with patients who are marginalized. Nurses’ working conditions, recognition, and understanding are strongly influenced by society, and calling nurses heroes undermines their capacity to challenge and resist the hero identity. The healthcare system’s impact on patient-nurse encounters challenges patients’ and nurses’ subjectivity, performativity, and power relations. The review allowed us to describe how Butler’s theoretical work can facilitate a reflection on nursing practice which is a prerequisite for caring, ethical relationships, and working conditions within a nursing context. Butler’s concepts can provide useful perspectives on how nurses understand, communicate with, and care for patients, as well as a nuanced understanding of the nursing role and power relations and structures.

## Introduction

Philosopher Judith Butler has influenced how people talk about vulnerable bodies and sees vulnerability as universal, existential, and relational. Being vulnerable is part of the human condition. The main theoretical areas that run across Butler’s work; power, knowledge and subjectivity, performativity, and ethics—are of particular relevance to nursing practice. This review aims to explore how Butler’s theoretical work is reflected in research literature within a nursing context.

## Background

The purpose of this review was to explore how the philosopher Judith Butler’s theoretical work is reflected in research literature within a nursing context and in relation to ethical concerns and practices. Butler’s post-structuralist, feminist, and ethical perspectives have been used to illuminate and discuss our way of living in a common world, political structures that challenge its inhabitants, and the value of the life of the other in a wide range of disciplines.^1–5^

Nagington^6^ has pointed out some concepts and perspectives from Butler that could be fruitful in nursing research and practice. He states that the themes of power, knowledge and subjectivity, performativity, and ethics are of particular relevance to nursing practice. Ethics and ethical relationships are some of the most used themes in Butler’s work, and she has examined ethics and ethical relationships from a variety of perspectives.^6^

Butler sees vulnerability as universal, existential, and relational. Her phenomenological view is that the lives of others are not our own, but they are connected to our life in the sense that our life, from the beginning, is dependent on a world of others, constituted in and by a social world.^7^

This perspective is connected to Butler´s view of human vulnerability as a fundamental condition. To be a human is always and already to be handed out to others, who can potentially also harm you. This fundamental vulnerability and dependence precedes subject formation, but it is also constitutive of the subject.^8^

Butler’s^9,10^ perspective is that we are introduced to the social world through our bodies. She is inspired by Merleau-Ponty’s phenomenology of the body, which takes the body as our point of departure for being in the world.^11^ The fundamental duality of the body shows that we both have a body and are a body, and are therefore, both a subject and an object. The duality of the body illuminates that our bodily existence in the world is, from the very beginning, intersubjective and social in that we are in the world and meet others who exist in the same way as ourselves. Butler.^9^ The body shapes through its belonging in its historical and cultural context, which makes a process of possibilities.^12^ Loss and vulnerability follow from our being socially constituted bodies. Being attached to others, we are at risk of losing those attachments, and being exposed to others, we are at risk of violence.^13^

Precarity is a central concept in Butler’s work that aims to point at a condition in which certain populations suffer from failing social and economic networks of support more than others, and become differentially exposed to injury, violence, and death.^11^ This condition is politically induced and points out that precarity as a lived reality is not an individual condition.^11^

For Butler, vulnerability and precarity are connected to our sociality, which is a fragile and necessary dimension of our interdependency. She argues for a conception of ethical obligations that is grounded in precarity,^7^ and connects this to the ethics of cohabitation in the global world of which we all are a part.

Butler’s work on relational non-violence, ethics, and politics is the fore questions concerning marginalized groups and individuals’ positions and experiences.^10^ Her writings revolve around the experiences of illegitimate or erased people and show how this way of thinking, with the aim of creating livable lives in the margins, can show the way to a better society for all.^8^ Butler’s book Precarious Life, the Powers of Mourning and Justice^13^ is based on her response to the events of 11 September 2001 and illuminates how the reactions after this incident dehumanized the Other by creating an us-them dichotomy. Butler argues that what can be said and shown is closely linked to which lives are worth living, what counts as a human life, and which deaths are mournable.

Butler’s theory of vulnerability and precarity also reveals how vulnerability can be a resource and a prerequisite for social communities, promoting livable lives for all.^9,13^ She points out that feeling the loss of the other makes us human. Inspired by the philosopher Emmanuel Levinas’ ethical philosophy and his notion of the “face of the other,” a conception of ethics that begins with the precarious life of the Other.^13^ Butler argues that the encounter with the other through face-to-face interactions is the fundamental occurrence from which our interdependence on collective and political social structures is derived.^7,13,14^ Encountering the “face of the other” and understanding its meaning serves as a reminder of the vulnerability and humanity of the other, the vulnerability of life itself.^15^ For vulnerability to be a productive force, she says, it must be pronounced, recognized, and acknowledged.^13^ Butler shows that vulnerability and precarity are closely linked to power.^15^

The concept of power is suffused in Butler’s analysis and is influenced by Foucault.^14,16^

For Foucault, power forms the subject, and is a central force in relations and encounters with other persons, knowledge, cultural context, governmental politics, and or different types of institutional structures.^13,16^ Power is understood as drawing boundaries for the subject’s freedom. Power is limiting but also enabling, which is the process Butler refers to as performativity.^6^

In her lecture “Performativity, precarity and sexual politics,” Butler^17^ described how the concept of precarity is connected to her core concept of gender performativity. To be a subject, one must first comply with certain norms that make a person recognizable.

Gender performativity paved the way for discussions of intersectionality. Gender is a performative, culturally and socially constructed identity and is the result of repeated performances, a series of imitations of what we associate with what is a “woman” and what is a “man.”^10^ The concept of performativity can be understood as something one does, an act, a verb in that it is a constant “doing” and at the same time also constantly being observed and understood by others in reference to discourses that are suffused with power and knowledge.^10,18^

Further, performativity is a concept that creates dynamism between discourse, power, the body, and subjectivity.^15^ Butler outlines how the body has a central place in politics in that it does something in the political field that words do not. Bodies are vulnerable in their encounters with violence and politics. She refers to people who are out in the streets demonstrating for a livable life as an example of how gathering in the streets to protest reveals how fragile physical security is. This shows how the gathering of people has a performative effect.^15^

Nagington^6^ suggests that the concept of performativity may help us understand how subjects must submit themselves to power and knowledge to engage in social relations.

According to Nagington,^6^ Butler’s work has facilitated critical reflection on the identities of patients, carers, and nurses and whether they always offer the best possibilities for ethical and caring relationships. Nagington^6^ points out that the application of Butler’s work in relation to nursing is in its infancy and suggests how Butler’s work can develop nursing research and practice.

## Aim

This paper aims to explore how Butler’s theoretical work is reflected in research literature within a nursing context.

## Methods

The framework described by Arksey and O’Malley^19^ and further refined by others^20,21^ was applied in this scoping review. The framework consists of the following steps: (1) identifying the research question, (2) identifying the relevant studies, (3) selecting studies, (4) charting the data, and (5) collating, summarizing, and reporting the results.

### Identifying relevant studies

The systematic search strategy and eligibility criteria were guided by the SPIDER framework (sample, phenomenon of interest, design, evaluation, and research type).^22^ The phenomenon of interest was inspired by Nagington^6^ article, which focuses on three areas of Butler’s theoretical work of particular relevance to nursing: 1; power, knowledge and subjectivity; 2 performativity, and 3; ethics. In addition, we searched for the phenomenon precarity and vulnerability which is central in Butler’s ethics Tables 1.^13^

**Table 1.** Inclusion and exclusion criteria using the sample, phenomenon of interest, design, evaluation, and research type (SPIDER) framework.

	Inclusion	Exclusion
Sample (S)	Papers including Judith Butler’s theoretical areas within nursing research and practice	Papers without Judith Butler’s Ethical theories/concepts
Phenomenon of interest (PI)	Papers related to nursing and Judith Butler’s themes: power, knowledge, and subjectivity; performativity; and ethics; and precarity and vulnerability	Papers related exclusively to Judith Butler’s other contexts than nursing.
Design (D)	All study designs	
Evaluation (E)	Butler’s theoretical areas reflected in research literature within a nursing context	
Research type (R)	All research types of studies published in Swedish, Danish, Norwegian, and English.	Grey literature, such as conference proceedings and abstracts, letters, comments editorials and non–peer-reviewed papers.
Context	Within a nursing context	All other aeries Exclusively nursing education
Time	No restriction on years	

In February 2023, a systematic search was performed in CINAHL (EBSCOhost), MEDLINE All (Ovid), Embase (Ovid), PsycINFO (Ovid), and Web of Science Core collection databases to identify relevant studies.

The search strategy was developed in MEDLINE (Ovid) by a research librarian (EKP) in collaboration with the first and last author (AHH & MEK) and was subsequently refined to make it as specific and sensitive as possible. The strategy comprised a single element, which included textwords for “Judith Butler” and terms related to the phenomenon of interest. Terms for conceptual constructs such as “theory,” “concept,” and “framework” in combination with “Butler” were also included to capture studies discussing Butler’s theories without explicitly mentioning them by name. The search terms were combined using both “AND” and “OR” Boolean operators to narrow down and expand the search and proximity operators were used to adjust sensitivity and specificity. To minimize the risk of bias, the search was not limited by combining the first element with a nursing-specific element, as it is difficult to cover nursing research in entirety. After reaching a consensus on the search strategy, the strategy was adapted for use in the other databases. In Web of Science, which covers a broad range of disciplines, including humanities and social sciences, the search was delimited to selected “research areas” related to medicine and health sciences (Appendix 1). Finally, the search strategy was peer reviewed by another research librarian in accordance with the Peer Review of Electronic Search Strategies (PRESS) guidelines.^23^ Search strategies are shown in Appendix 2. The first and last author (AHH & MEK) performed manual searches to screen the reference lists of the included papers.

### Selecting studies

**Figure 1.**
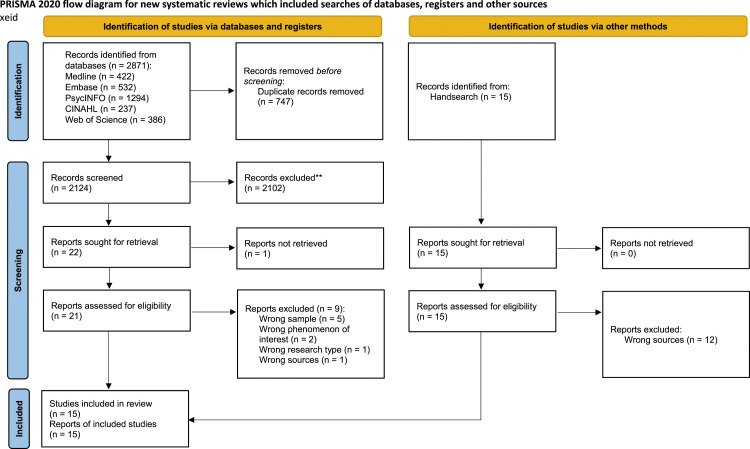
PRISMA 2020 flow diagram for new systematic reviews which included searches of databases, registers, and other sources. *Consider, if feasible to do so, reporting the number of records identified from each database or register searched (rather than the total number across all databases/registers). **If automation tools were used, indicate how many records were excluded by a human and how many were excluded by automation tools. From: Page MJ, McKenzie JE, Bossuyt PM, Boutron I, Hoffmann TC, Mulrow CD, et al. The PRISMA 2020 statement: an updated guideline for reporting systematic reviews. BMJ 2021; 372: n71. DOI: 10.1136/bmj.n71. For more information, visit: https://www.prisma-statement.org/.

The database searches returned a total of 2871 references, which were subsequently imported into the EndNote software and de-duplicated using Bramers’ method by the research librarian.^24^ After removing duplicates, 2124 references were randomly assigned to three groups and uploaded to the web application Rayyan to facilitate blinding.^25^ Three pairs of authors (AHH & MEK, EKP & MHI, MCR & TLJ) independently assessed the titles and abstracts to determine their suitability for inclusion in the study. Following a full-text screening of 21 papers by the same author pairs, 12 papers were deemed eligible for inclusion in the review. Three additional articles were included following the manual search. Any discrepancies or disagreements between the pairs were resolved through a collective meeting involving all authors, with the final resolution being carried out by first and last author. The study selection process is shown in the PRISMA 2020 flow diagram (Figure 1).

### Charting the data

According to Levac, Colquhoun 21 data charting should be viewed as an iterative process, in which the data charting form is subject to continuous updates. Two of the authors (AHH and MEK) developed a data charting form for the purpose of data extraction. Following the full-text screening, the charting form was reviewed and discussed by the research team and subsequently modified by the same two authors who initially developed it to ensure its suitability with respect to the data and research question.

### Collating, summarizing, and reporting results

We summarized the entire dataset in terms of authors, publication year, journal, title, country of origin, publication type, aim, methods, findings or results, and description of Judith Butler’s themes and references to Butler’s work. During the analysis process, the authors continuously reflected on the findings and discussed their implications.

### Consult with the reference group

We consulted with stakeholders in the research group to examine our approach to the review and to validate the findings in line with Arksey and O’Malley^19^ recommendation to enhance the meaning and applicability of the scoping review.

## Results

The included 15 articles consisted of six theoretical papers, one discussion paper, and eight empirical research papers. Geographically, studies were from Canada (6), the UK (6), Chile (1), and the USA (2). The papers were published between 2002 and 2022.

All included papers were reviewed for explicit descriptions of Butler’s theoretical perspectives: power, knowledge, and subjectivity; performativity; ethics; and precarity and vulnerability. Performativity was used in seven papers,^6,26–31^ subjectivity in seven,^6,29,31–35^ precarity in one,^36^ power and power relations in five,^6,32,33,35,36^ the concept of norms in two,^37,38^ gender in two,^6,38^ and vulnerability in one.^39^

Table 2 Butler’s theories were applied at an individual and social level in nursing research and practice. The included 15 papers revolved around three main themes: nursing practice and impact related to marginalized patient groups, nurses’ working conditions, recognition and understanding are influenced by society, and the healthcare system’s impact on patient-nurse encounters.

**Table 2.** Overview of the included articles.

Author(s)	Year	Country	Journal	Aim/Purpose	Judith Butler’s themes	Research type	References
Aston, M., Price, S., Kirk, S. F. L. & Penney, T.	2011	Canada	Journal of Advanced Nursing	To discuss the application of a feminist poststructuralist-based theoretical framework as an approach towards understanding and managing the complex health issue of obesity	Subjectivity, power relations, agency	Discussion paper	Butler, J. (1992) Contingent foundations: Feminism and the question of “postmodernism.” In J. Butler & J. Scott (Eds.), *Feminists Theorize the Political* (pp. 3-21). Routledge. Butler J. (2005) *Giving an Account of Oneself*. Fordham University Press.
Aston, M. L.(håndsøk)	2002	Canada	Public Health Nursing	The paper discusses how social structures of isolation, investment in a medical discourse, and processes of normalization construct an individual’s experiences and practices of mothering, which in turn influence pedagogical practices in postpartum classes	Subjectivity	Empirical paper	Butler, J. (1992). Contingent foundations: Feminism and the question of “postmodernism.” In J. Butler & J. Scott (Eds.). Feminists *theorize the political.* Routledge
Aston, M., Meagher-Stewart, D., Sheppard-Lemoine, D., Vukic, A. & Chircop, A.	2006	Canada	Pediatric Nursing	To examine how empowerment, as an ideology and a practice of teaching and learning, was understood and applied by public health nurses in health education with childbearing and child-rearing families	Subjectivity, power relations, agency	Empirical paper Pilot study	Butler, J. (1992). Contingent foundations: feminism and the question of “postmodernism.” In J. Butler & J. Scott (Eds.). Feminists *theorize the political.* Routledge
Dewart, G., Estefan, A., Clandinin, D. J. & Caine, V.	2021	Canada	Research & Theory for Nursing Practice	To explore waiting as a relational, formative process, drawing on experiences from women who disclosed substance use during pregnancy and prenatal period.	Performativity	Empirical paper	Butler, J. (1988). Performative acts and gender constitution: An essay in phenomenology and feminist theory. *Theatre Journal* 40(4): 519–531. Butler, J. (1990). Gender Trouble. Routledge.
Hales, S. & Tyler, M.	2022	UK	Gender, Work & Organization	The paper draws on Butler’s writing on precarity and the interpellatory power of naming and reads through her writing on the dynamics of recognition, vulnerability, and resistance, to develop a critique of the discourse of heroism used as a position health and social care professionals, and other keyworkers during the Covid pandemic.	Precarity, power	Theoretical paper	Butler, J. (1997). *Excitable Speech: A Politics of the Performative*. Routledge. Butler, J. (2000). *Gender Trouble*. Routledge. Butler, J. (2009). *Frames of War*. Verso books. Butler, J. (2015). *Notes Towards a Performative Theory of Assembly*. Harvard university Press. Butler, J. (2016). Rethinking vulnerability and Resistance. In J. Butler, Z. Gambetti & L. Sabsay (Eds.), *Vulnerability in Resistance* (pp. 12–27). Duke university press. Butler, J. (2021). Recognition and the social bond. In H. Ikäheimo, K. Lepold & T. Stahl (Eds.), *Recognition and Ambivalence* (pp. 31–53). Columbia: University Press. Butler, J. & Athanasiou, A. (2013). *Dispossession: The Performative in the Political*. Cambridge: Polity.
Madariaga, F. A. C., Garcés, B. E. G., Parra, A. C. & Foster, J.	2017	Chile	Nursing Inquiry	To investigate the experiences of 10 women in the female central penitentiary of Santiago, Chile, regarding their sexuality within prison. To explore how midwives and nurses can promote healthy sexuality for incarcerated women.	Performativity	Empirical paper	Butler, J. (1990). *Gender trouble: Feminism and the subversion of identity*. Routledge. Butler, J. (1993). *Bodies that matter: On the discursive limits of sex.* Routledge.
Mäkelä, P.	2018	UK	Journal of Evaluation in Clinical Practice	To explore the negotiation of nursing home residents’ transfers to hospital, where they may have been avoidable.	Performativity	Theoretical paper	Butler, J. (1993). *Bodies that matter: On the discursive limits of sex*. Routledge. Butler, J. (1990). *Gender trouble: Feminism and the subversion of identity.* Routledge. Butler J. (2010). Performative agency. *Journal of Cultural Economy 3*(2): 147–161
McDonald, J.	2013	USA	Gender, Work & Organization	To produce significant insights on the gender performances of nursing students by exploring the diverse ways through which male and female nursing students make sense of their gender identities and their performances.	Gender norms	Empirical paper	Butler, J. (1993). *Bodies that Matter: On the Discursive Limits of Sex*. Routledge. Butler, J. (2004). *Undoing Gender*. Routledge. Butler, J. (2007). *Gender Trouble: Feminism and the Subversion of Identity*. Routledge.
Nagington, M. G.	2016	UK	Nursing Philosophy	To explore the works and theories of Judith Butler, and how her theories could be used in nursing research.	Power, (knowledge, subjectivity gender) performativity, ethics	Theoretical paper (with empiric examples.)	Butler, J. (1990). *Gender Trouble: Feminism and the Subversion of Identity*. Routledge. Butler, J. (1993). *Bodies That Matter: On the Discursive Limits of sex*. Routledge. Butler, J. (1997a). *Excitable Speech: A Politics of the Performative*. Routledge. Butler, J. (1997b). *The Psychic Life of Power: Theories in Subjection*. Stanford university Press. Butler, J. (2004a). *Precarious Life: The Powers of Mourning and Violence*. Verso. Butler, J. (2004b). *Undoing gender*. Routledge. Butler, J. (2005). *Giving an Account of Oneself*. Fordham university Press. Butler, J. (2009). *Frames of war: When is Life Grievable?* Verso. Butler, J. & Salih, S. (2004). *The Judith Butler Reader*. Blackwell.
Nagington, M., Luker, K. & Walshe, C.	2013	UK	Nursing Ethics	To explore the views of patients and their caregivers in the context of palliative and supportive district nursing care, and to explore how sociopolitical ideologies affect the quality and morality of care.	Subjectivity, performativity	Empirical paper	Butler, J. (1997). *The psychic life of power: Theories in subjection*. Stanford university Press.
Nagington, M., Walshe, C. & Luker, K. A.	2016 a	UK	Nursing Philosophy	The ethical implications of the ways in which technology shapes the subjectivities of patients and carers.	Subjectivity, power	Empirical paper.	Butler, J. (1990). *Gender Trouble: Feminism and the Subversion of Identity*. Routledge, London. Butler, J. (1993). *Bodies that Matter: On the Discursive Limits of Sex*. Routledge. Butler, J. (1997a). *Excitable Speech: A Politics of the Performative*. Routledge. Butler, J. (1997b). *The Psychic Life of Power: Theories in Subjection*. Stanford university Press. Butler, J. (2004). *Precarious Life: The Powers of Mourning and Violence*. Verso. Butler, J. (2005). *Giving an Account of Oneself.* Fordham university Press.
Nagington, M., Walshe, C. & Luker, K. A.	1016 b	UK	Nursing *Inquiry*	To examine how knowledge as an ethical concept impinges upon quality of care and how quality of care is assessed and produced in healthcare	Subjectivity, performativity	Empirical paper	Butler, J. (1990). *Gender Trouble: Feminism and the Subversion of Identity*. Routledge, London. Butler, J. (1997a). *Excitable Speech: A Politics of the Performative*. Routledge. Butler, J. (1997b). *The Psychic Life of Power: Theories in Subjection*. Stanford university Press. Butler, J. (2005). *Giving an Account of Oneself.* Fordham university Press.
Petrovskaya, O., McDonald, C. & McIntyre, M.	2011	Canada	Nursing Philosophy	To recognize and disrupt the forces of the research industry with its instrumental reason in graduate nursing education	Norms	Theoretical paper	Butler, J. (2004). *Undoing Gender*. Routledge.
Phillips, C. R.	2021	Canada	Qualitative Social Work	Examine the juncture and relationship between vulnerability and heroism.	Vulnerability	Theoretical paper	Butler, J. (2004). *Precarious Life: The Powers of Mourning and Violence*. Verso.
Phillips, D. A., Phillips, K. H., Grupp, K. & Trigg, L. J.	2009	USA	Advances in Nursing Science	To shed light on sibling violence and why nurses should be on the front lines of ending such practice.	Performativity	Theoretical paper	Butler, J. (1997). *Excitable Speech: A Politics of the Performative.* Routledge. Butler, J. (1993). *Bodies that Matter: On the Discursive Limits of Sex*. Routledge.

### Nursing practice and impact related to marginalized patient groups

This scoping review revealed that nurses’ performativity, connected to their norms and values and the everyday organization of their work with patients, affected patients and created more vulnerable situations for them than necessary. Six papers examine marginalized patient groups that are particularly vulnerable, such as obese patients,^32^ female substance users with newborn children,^26^ female prisoners,^27^ nursing home residents in transition to the hospital,^28^ patients receiving palliative care from a district nurse,^6^ and children with a violent sibling.^30^ These papers show that nurses need to reflect on and examine their clinical practice and role as health professionals in relation to power and performativity in encounters with patients who are marginalized. Using Butler’s theories, the papers discuss the ways in which healthcare professionals can reflect on and analyze institutional and social influences on their practice^6,26,32^ by questioning “normal” or everyday practices and identifying tensions, conflicts, or differences. It is essential to acknowledge the performativity in these acts that patients, clients, and their families do to preserve themselves in challenging encounters and living situations, agency, and identity work.^26,28,38^ To permit greater reflexivity in nursing practice and to allow nurses to become aware of power relations situated within social structures of meaning and the power relations the care providers hold becomes essential in good nursing practice. Phillips and Phillips^30^ also point out that discussing sibling violence is a social justice issue that healthcare professionals must consider.

### Nurses’ working conditions, recognition, and understanding are influenced by society

Two of the papers discuss society’s labeling of nurses as heroes during the COVID-19 pandemic in light of Butler’s theories of precarity, power, and vulnerability^36,39^ and how society’s view undermines nurses’ capacity to challenge and resist the hero positioning.^36^ The concept of heroism is ambiguous. Nurses are vulnerable in their working situation, despite their heroism, which obscures the fact that they are also ordinary citizens who must cope with their everyday lives. Society’s expression of gratitude through clapping, rainbows, and hearts contributes to this perception of heroism and makes vulnerability unrecognizable.^39^ Nursing is influenced by societal norms, starting in nursing education. How nursing education is developed will affect nursing values and norms. Therefore, teaching students to be aware of and expose power relations and norms is crucial.^37^

The occupation of nursing as gendered with feminine cultural norms and not as an occupation where nursing students, both male and female in their training, may challenge the gendered norms in developing nursing identity.^38^

### The healthcare system’s impact on the patient-nurse relationship

Three papers^6,28,29^ draw attention to how the healthcare system and its organization challenge patients’ and nurses’ subjectivity, performativity, and power relations. Mäkelä^28^ discusses transfers of nursing home residents to the hospital that could have been avoided if the focus was on the residents’ needs (person-centered care) and the nursing staff could make decisions rather than follow organizational and functional requirements. Nagington^29^ shows how nurses’ busyness shapes and limits palliative patients’ and carers’ subjectivity to home-based care quality. They become docile in relation to the “busy” nurse. The “busyness” of the district nurse must be seen in connection with the health political vision of providing more efficient home-based care.

Nagington et al.^32^ show how technology affects palliative patients and their families in their own homes and shapes how patients and carers may reclaim their power over their homes. To make the home viable location for providing care, we may consider the opportunities technology provides to become part of the home instead of the home becoming a hospital.

Nagington et al.^31^ explored the ways in which knowledge influences nurse-patient-carer relationships in palliative and supportive district nursing care, using Butler’s concepts of subjectivity and performativity. Their results show that patients in palliative care and their carers had no idea of what district nurses could do for them or how they could support them beyond the care they were receiving. The lack of knowledge of what district nurses do beyond tasks such as helping with basic needs and providing information about disease and illness, prevented patients and carers from learning about the possibility of district nursing services, such as psychological support and or how district nurses could be involved with potential future care needs. Due to geographical isolation and the fact that the care and treatment are conducted at home, patients and carers have few opportunities to get information about district nursing through networking with other patients and carers. As a result, they have little possibility to influence or resist the care they get from the district nurse because the only one to change the structure of this care or the acts performed would be the district nurse.

Aston^35^ and Aston et al.^34^ discuss the complexity of the relationship between public family health nurses (PHNs) and mothers with newborn babies in postpartum groups and in-home visits in empowering the mothers through knowledge exchange. Aston^35^ used Butler’s concepts of agency and power relations to show that PHNs’ pedagogical practices and beliefs about learning and mothering were complex and could also be contradictory. The mothers had their own beliefs, mothering practices, and understanding of the postpartum group’s purpose, which challenged the PHNs’ intention to facilitate a supportive group with the attending mothers. Applying Butler’s concept of agency and power relations, Aston et al.^34^ found that individual choice and recognition of knowledge and power informed how both PHNs and mothers used their “agency” to position themselves within a particular relationship.

## Discussions

In the reviewed papers, we found that Butler’s theoretical perspectives were applied at an individual and social level to research literature in a nursing context. The included papers revolved around three main themes: nursing practice and impact related to marginalized patient groups; nurses’ working conditions, recognition, and understanding are influenced by society; and the healthcare system’s impact on patient-nurse encounters.

The papers focused on marginalized patient groups that are in a particularly vulnerable condition and highlighted that nurses’ performativity in relation to their norms and values could lead to more vulnerable situations for patients. Being vulnerable is part of the human condition. However, Sellman^40^ explained that people who are or become recipients of health care in general and nursing care in particular can be considered more-than-ordinarily vulnerable. Nurses need to reflect on and have a critical view of their clinical practice and role as health professionals in relation to power and performativity in encounters with patients who are marginalized by societies’ norms and values, and their ways of living. Other studies emphasized that patients’ vulnerability is a key issue in nursing^41,42^ and that a protective function is a fundamental part of the role of nurses.^40^

The understanding of our common vulnerability as humans may also be important to consider and is central to nursing ethics. Purdy^43^ highlighted that the essence of being vulnerable is openness to circumstances and the foundation of being affected. Butler’s theoretical perspectives on precarity, power, and vulnerability were used in papers that examined nurses’ own precarity and vulnerability during the COVID-19 pandemic. As the COVID-19 pandemic spread over the world, nurses feared for their own and their family’s health and to bring the disease to their patients, which contrasted with their image as heroes. Calling nurses heroes undermines their ability to reveal their own vulnerability, which is discussed in this review in relation to precarity, power, and vulnerability. A recent study by Hillestad et al.^44^ found that the ideology of person-centered care and the societies’ ideology of a nursing home as a home for the residents were, therefore, nearly impossible to manage when the needs of society had to be prioritized over the individual resident.

In this review, we found papers calling for healthcare systems to take responsibility for nurses’ working conditions. Nurses’ recognition and understanding are influenced by society and patient-nurse subjectivity, performativity, and power relations. Using the concept of performativity, several of the papers highlighted how nursing treatment and care, institutional architecture, nurses’ uniforms, or how we speak can create stress instead of contributing to the desired treatment and care. Patients with obesity, for example, or who, due to their life situation, excrete a bad smell can challenge nurses treatment and care, which in turn is expressed through the nurses’ bodily postures and behavior.

This review shows that factors that intertwine and have an impact on nurses’ encounters with patients and how their work is organized because nurses are also a part of society. Nurses’ attitudes toward, for instance, patients who are obese, drug addicts, or prisoners may affect the nurse-patient encounter. Therefore, nurses’ attitudes and values will reflect society’s attitudes through the healthcare system’s structure and policy.

Nagington et al.^29^ and Nagington^6^ showed Butler’s perspective on performativity can contribute to a broader understanding of how nurse-patient encounters are linked to social and cultural factors and how these factors may be influenced by power relations and knowledge; in this way, nurses are challenged to scrutinize how they refer to groups and individuals and how concepts such as health, normality, and outsiders are used and defined so that vulnerability can come to the fore.

Nagington^6^ suggested how Butler’s work can be used to develop nursing and highlights her perspective on gender. However, Butler didn’t reveal the importance of gender in relation to nursing. McDonald^38^ discusses gender norms in nursing and how nursing students do and undo learning of gender in nursing education. How we learn and practice nursing and how society understands nursing have previously been linked to nursing’s status as a women’s profession, as something feminine because it involves, among other things, tasks of caring. Nevertheless, it would appear that society’s understanding of nursing is still that nurses “sacrifice themselves,” which is why, for example, they become heroes and are applauded when they are at the front of, for example, the COVID-19 pandemic. Butler’s perspectives on gender and gender performativity can be used to reflect on nursing and nursing practice, for instance, creating a space for both patients and colleagues to express their gender without being stigmatized, and the feminine gender performance in nursing discussed in this section. Butler’s perspectives may help increase nurses’ awareness of their relationship to society, the economy, and politics and improve everyday life for both patients and nursing practice.

Butler’s gender performativity has formed the basis for the development of their preoccupation with ethics and non-violent politics.^6^ Butler is inspired by Merleau-Ponty’s phenomenology of the body and an understanding of the body as fundamental to being in the world and having relationships. Moreover, the body is shaped through a lived life in a historical, cultural, and social context and makes humans vulnerable in different situations and relationships. The individual’s bodily history, therefore, becomes essential in nursing practice.

This review doesn’t show Butler’s phenomenological perspective on the body directly but more implicitly through the vulnerability of the body that is marginalized in the encounter with nurses and the healthcare system.^26–28,30,32,33^

During the COVID-19 pandemic, nursing in a global context was brought up to date, although this review does not address this issue. In relation to Butler’s^9^ preoccupation with being able to live livable lives, she discusses how vulnerability to COVID-19 disease and access to vaccines were unequally distributed in the world. The International Council of Nurses (ICN) emphasizes that a nurse’s role is to be an advocate for equality and social justice in resource allocation, access to health care, and other social and economic services, as well as to actively support others in speaking for themselves or speaking on behalf of others who cannot speak for themselves.^45^

Braidotti stated that there is a common concern about the health of both humans and animals as a result of climate change, urbanization, wars and terrorism, and microbial and chemical pollution of land and water sources.^46^ The ICN emphasizes that nurses collaborate and practice to preserve, sustain, and protect the natural environment and are aware of the health consequences of environmental degradation, such as climate change. Nurses advocate for initiatives that reduce environmentally harmful practices to promote health and well-being.^45^ Butler’s reflections in relation to vulnerability and exposure and a livable life in regard to climate change and its effects globally may be an important contribution to nursing research on individual, institutional, and societal levels. Butler points out that if we, as humans, inhabit the earth without regard for biodiversity and without stopping climate change, then we produce an uninhabitable world that will have a big impact on our way of living and where in the world it is possible to have a livable life.^9^ This will have consequences for nursing at both a local and a global level.

### Strength and limitations

To enhance the review findings’ relevance for nursing and nursing practice, we conducted a consultation exercise. We conducted the scoping review in line with an acknowledged methodological framework and analysis methods. To ensure transparency, the review process has been described in detail.^19–21^ The search strategy was peer reviewed by another research librarian in accordance with the Peer Review of Electronic Search Strategies (PRESS) guidelines.^23^

We did not appraise the quality of evidence of the included studies but rather presented how Butler’s theoretical work is reflected in them. In addition, as our review had some language limitations (English and Scandinavian), there may be studies that we were unable to identify. We acknowledge that this review may not have captured all relevant materials as we did not include grey literature here defined as “information produced on all levels of government, academics, business, and industry in print and electronic formats, but which is not controlled by commercial publishers”.^47^

## Conclusions

In this review, we have described how Butler’s theoretical work can facilitate a critical reflection on nursing practice which is a prerequisite for caring, ethical relationships, and working conditions in a nursing context. Butler’s perspectives can offer insight into how nurses understand, communicate with, and care for patients, as well as a nuanced understanding of the nursing role, power relations, and structures. Using Butler’s understanding of the body more explicitly in nursing research would further help to illuminate vulnerability and ethical challenges in nursing practice. Furthermore, Butler’s perspectives illuminate how global health influences local nursing practice.

## Supplemental Material


Supplemental Material - Judith Butler’s theoretical perspectives within a nursing context—a scoping review
Supplemental Material for Judith Butler’s theoretical perspectives within a nursing context—a scoping review by Adelheid Hummelvoll Hillestad, Eline Kaupang Petersen, Maud C Roos, Maria H Iversen, Trine Lise Jansen, and Monica Evelyn Kvande in Journal of Nursing Ethics.

Supplemental Material - Judith Butler’s theoretical perspectives within a nursing context—a scoping review
Supplemental Material for Judith Butler’s theoretical perspectives within a nursing context—a scoping review by Adelheid Hummelvoll Hillestad, Eline Kaupang Petersen, Maud C Roos, Maria H Iversen, Trine Lise Jansen, and Monica Evelyn Kvande in Journal of Nursing Ethics.
