# Supplementary material for: Judith Butler’s theoretical perspectives within a nursing context—a scoping review
Source: Nurs Ethics. 2024 Jun 5;32(1):288–305. doi: 10.1177/09697330241257569 (PMC11771098; doi:10.1177/09697330241257569)
Supplement: Supplemental Material - Judith Butler’s theoretical perspectives within a nursing context—a scoping review [file sj-pdf-1-nej-10.1177_09697330241257569.pdf]

Appendix 1: List of selected 'Research Areas'-limiters in Web of Science

- Psychology
- Science Technology Other Topics
- Biochemistry Molecular Biology
- Physiology
- Public Environmental Occupational Health
- Social Work
- Biomedical Social Sciences
- Psychiatry
- Nursing
- Dentistry Oral Surgery Medicine
- Life Sciences Biomedicine Other Topics
- Health Care Sciences Service
- Neurosciences Neurology
- Biotechnology Applied Microbiology
- Evolutionary Biology
- Genetics Heredity
- Rehabilitation
- Cell Biology
- Endocrinology Metabolism
- Biophysics
- Cardiovascular System Cardiology
- General Internal Medicine
- Pharmacology Pharmacy
- Radiology Nuclear Medicine Medical Imaging
- Substance Abuse
- Surgery
- Urology Nephrology
- Research Experimental Medicine
- Geriatrics Gerontology
- Legal Medicine
- Nutrition Dietetics
- Oncology
- Pathology
- Pediatrics
- Medical Informatics
- Microbiology
- Parasitology
- Transplantation
- Tropical Medicine
- Anatomy Morphology
- Anesthesiology
- Audiology Speech Language Pathology
- Behavioral Sciences

- Dermatology
- Medical Ethics
- Ophthalmology
- Orthopedics
- Respiratory System
- Toxicology
