# Supplementary material for: Judith Butler’s theoretical perspectives within a nursing context—a scoping review
Source: Nurs Ethics. 2024 Jun 5;32(1):288–305. doi: 10.1177/09697330241257569 (PMC11771098; doi:10.1177/09697330241257569)
Supplement: Supplemental Material - Judith Butler’s theoretical perspectives within a nursing context—a scoping review [file sj-pdf-2-nej-10.1177_09697330241257569.pdf]

Appendix 2: Search strategies

Database: Ovid MEDLINE(R) ALL

#	Query
**1**	("judith butler*" or "butler, j" or "butler,j" or "butler, judith" or "j. butler*" or "j.butler*" or butlerian*).tw,kf.
**2**	(butler* adj6 theor*).tw,kf.
**3**	(butler* adj6 concept*).tw,kf.
**4**	(butler* adj6 (work or works)).tw,kf.
**5**	(butler* adj6 framework*).tw,kf.
**6**	(butler* adj6 notion*).tw,kf.
**7**	(butler* adj6 (idea or ideas)).tw,kf.
**8**	(butler* and vulnerab*).tw,kf.
**9**	(butler* and precar*).tw,kf.
**10**	(butler* and perform*).tw,kf.
**11**	(butler* and ethic*).tw,kf.
**12**	(butler* and subject*).tw,kf.
**13**	(butler* and power*).tw,kf.
**14**	(butler* and knowledge*).tw,kf.
**15**	(butler* and nurs*).tw,kf.
**16**	1 or 2 or 3 or 4 or 5 or 6 or 7 or 8 or 9 or 10 or 11 or 12 or 13 or 14 or 15

Database: Ovid Embase

#	Query
**1**	("judith butler*" or "butler, j" or "butler,j" or "butler, judith" or "j. butler*" or "j.butler*" or butlerian*).tw,kf.
**2**	(butler* adj6 theor*).tw,kf.
**3**	(butler* adj6 concept*).tw,kf.
**4**	(butler* adj6 (work or works)).tw,kf.
**5**	(butler* adj6 framework*).tw,kf.
**6**	(butler* adj6 notion*).tw,kf.
**7**	(butler* adj6 (idea or ideas)).tw,kf.
**8**	(butler* and vulnerab*).tw,kf.
**9**	(butler* and precar*).tw,kf.
**10**	(butler* and perform*).tw,kf.
**11**	(butler* and ethic*).tw,kf.
**12**	(butler* and subject*).tw,kf.
**13**	(butler* and power*).tw,kf.
**14**	(butler* and knowledge*).tw,kf.
**15**	(butler* and nurs*).tw,kf.
**16**	1 or 2 or 3 or 4 or 5 or 6 or 7 or 8 or 9 or 10 or 11 or 12 or 13 or 14 or 15

Database: Ovid PsycINFO

#	Query
**1**	("judith butler*" or "butler, j" or "butler,j" or "butler, judith" or "j. butler*" or "j.butler*" or butlerian*).tw.
**2**	(butler* adj6 theor*).tw.
**3**	(butler* adj6 concept*).tw.
**4**	(butler* adj6 (work or works)).tw.
**5**	(butler* adj6 framework*).tw.
**6**	(butler* adj6 notion*).tw.
**7**	(butler* adj6 (idea or ideas)).tw.
**8**	(butler* and vulnerab*).tw.
**9**	(butler* and precar*).tw.
**10**	(butler* and perform*).tw.
**11**	(butler* and ethic*).tw.
**12**	(butler* and subject*).tw.
**13**	(butler* and power*).tw.
**14**	(butler* and knowledge*).tw.
**15**	(butler* and nurs*).tw.
**16**	1 or 2 or 3 or 4 or 5 or 6 or 7 or 8 or 9 or 10 or 11 or 12 or 13 or 14 or 15

Database: CINAHL Complete via EBSCO Host

#	Query	Limiters/Expanders
**S1**	TI("judith butler*" or "butler, j" or "butler,j" or "butler, judith" or "j. butler*" or "j.butler*" or butlerian*) OR AB("judith butler*" or "butler, j" or "butler,j" or "butler, judith" or "j. butler*" or "j.butler*" or butlerian*)	Search modes - Boolean/Phrase
**S2**	TI(butler* N5 theor*) OR AB(butler* N5 theor*)	Search modes - Boolean/Phrase
**S3**	TI(butler* N5 concept*) OR AB(butler* N5 concept*)	Search modes - Boolean/Phrase
**S4**	TI(butler* N5 (work or works)) OR AB(butler* N5 (work or works))	Search modes - Boolean/Phrase
**S5**	TI(butler* N5 framework*) OR AB(butler* N5 framework*)	Search modes - Boolean/Phrase
**S6**	TI(butler* N5 notion*) OR AB(butler* N5 notion*)	Search modes - Boolean/Phrase
**S7**	TI(butler* N5 (idea or ideas)) OR AB(butler* N5 (idea or ideas))	Search modes - Boolean/Phrase
**S8**	TI(butler* and vulnerab*) OR AB(butler* and vulnerab*)	Search modes - Boolean/Phrase
**S9**	TI(butler* and precar*) OR AB(butler* and precar*)	Search modes - Boolean/Phrase
**S10**	TI(butler* and perform*) OR AB(butler* and perform*)	Search modes - Boolean/Phrase
**S11**	TI(butler* and ethic*) OR AB(butler* and ethic*)	Search modes - Boolean/Phrase
**S12**	TI(butler* and subject*) OR AB(butler* and subject*)	Search modes - Boolean/Phrase
**S13**	TI(butler* and power*) OR AB(butler* and power*)	Search modes - Boolean/Phrase
**S14**	TI(butler* and knowledge*) OR AB(butler* and knowledge*)	Search modes - Boolean/Phrase
**S15**	TI(butler* and nurs*) OR AB(butler* and nurs*)	Search modes - Boolean/Phrase
**S16**	S1 OR S2 OR S3 OR S4 OR S5 OR S6 OR S7 OR S8 OR S9 OR S10 OR S11 OR S12 OR S13 OR S14 OR S15	Search modes - Boolean/Phrase

Database: Web of Science via Clarivate – Web of Science Core Collection

#	Query
**1**	TS=("judith butler*" or "butler, j" or "butler,j" or "butler, judith" or "j. butler*" or "j.butler*" or butlerian*)
**2**	TS=(butler* NEAR/5 theor*)
**3**	TS=(butler* NEAR/5 concept*)
**4**	TS=(butler* NEAR/5 (work or works))
**5**	TS=(butler* NEAR/5 framework*)
**6**	TS=(butler* NEAR/5 notion*)
**7**	TS=(butler* NEAR/5 (idea or ideas))
**8**	TS=(butler* and vulnerab*)
**9**	TS=(butler* and precar*)
**10**	TS=(butler* and perform*)
**11**	TS=(butler* and ethic*)
**12**	TS=(butler* and subject*)
**13**	TS=(butler* and power*)
**14**	TS=(butler* and knowledge*)
**15**	TS=(butler* and nurs*)
**16**	#15 OR #14 OR #13 OR #12 OR #11 OR #10 OR #9 OR #8 OR #7 OR #6 OR #5 OR #4 OR #3 OR #2 OR #1
**17**	Limited to the following 'research areas':<br>• Psychology<br>• Science Technology Other Topics<br>• Biochemistry Molecular Biology<br>• Physiology<br>• Public Environmental Occupational Health<br>• Social Work<br>• Biomedical Social Sciences<br>• Psychiatry<br>• Nursing<br>• Dentistry Oral Surgery Medicine<br>• Life Sciences Biomedicine Other Topics<br>• Health Care Sciences Service<br>• Neurosciences Neurology<br>• Biotechnology Applied Microbiology<br>• Evolutionary Biology<br>• Genetics Heredity<br>• Rehabilitation

- Cell Biology
- Endocrinology Metabolism
- Biophysics
- Cardiovascular System Cardiology
- General Internal Medicine
- Pharmacology Pharmacy
- Radiology Nuclear Medicine Medical Imaging
- Substance Abuse
- Surgery
- Urology Nephrology
- Research Experimental Medicine
- Geriatrics Gerontology
- Legal Medicine
- Nutrition Dietetics
- Oncology
- Pathology
- Pediatrics
- Medical Informatics
- Microbiology
- Parasitology
- Transplantation
- Tropical Medicine
- Anatomy Morphology
- Anesthesiology
- Audiology Speech Language Pathology
- Behavioral Sciences
- Dermatology
- Medical Ethics
- Ophthalmology
- Orthopedics
- Respiratory System
- Toxicology
